# Preventive effects of early mobilisation on delirium incidence in critically ill patients: systematic review and meta-analysis

**DOI:** 10.1007/s00063-024-01243-8

**Published:** 2025-03-14

**Authors:** Li Zhou, Fei Xie, Yangyang Zeng, Xi Xia, Rui Wang, Yongjie Cai, Yu Lei, Fengjiao Xu, Xi Li, Bing Chen

**Affiliations:** 1 Department of Intensive Care, The People’s Hospital of Leshan, 614000 Leshan, China; 2 Department of Surgery, Mianyang People’s Hospital, 621053 Mianyang, China; 3 Department of Neurosurgery, The People’s Hospital of Leshan, 614000 Leshan, China; 4 Department of Nursing, The People’s Hospital of Leshan, No. 238 Baita Street, Shizhong District, 614000 Leshan, China

**Keywords:** Early mobilisation, Delirium, Non-drug intervention, Intervention, Rehabilitation, Eine frühe Mobilisierung, Geistige Verwirrung, Nichteinnahme von Medikamenten, Ich gehe nicht zur Polizei, Genesung

## Abstract

**Objective:**

To evaluate whether early mobilisation can reduce the incidence of delirium in critically ill patients and to assess the methodological quality of published studies.

**Methods:**

Three electronic databases, PubMed, Embase and the Cochrane Library, were searched for relevant studies published up to 2 March 2024. Articles were screened independently by two reviewers, based on inclusion and exclusion criteria, and a meta-analysis was performed using RevMan 5.3 software with a random-effects model.

**Results:**

A total of 18 studies (intervention group: 1794 participants, control group: 2129 participants) were included in the systematic review, with 18 studies included in the meta-analysis. Early mobilisation was found to reduce the risk of delirium in critically ill populations, with a pooled odds ratio of 0.65 (95% confidence interval [CI] 0.49–0.86; *P* = 0.003; I^2^ = 59%). Additionally, two studies found that early mobilisation did not change the duration of delirium in critically ill populations, with a pooled mean difference of −1.53 (95% CI −3.48 to 0.41; *P* = 0.12; I^2^ = 37%). Subgroup analysis revealed that early mobilisation maintained its preventive effect on delirium in the before/after intervention studies, studies published before 2018 and studies with a moderate methodological rating.

**Conclusion:**

As a nonpharmacological intervention, early mobilisation may help reduce the risk of delirium and shorten its duration in critically ill patients compared with standard treatment and may potentially become a novel strategy for delirium prevention in future intensive care unit settings.

**Supplementary Information:**

The online version of this article (10.1007/s00063-024-01243-8) contains supplementary material, which is available to authorized users.

## Abstract (de)

**Zielsetzung:**

Es sollte untersucht werden, ob eine frühzeitige Mobilisierung die Häufigkeit von Delirien bei kritisch kranken Patienten verringern kann. Ferner sollte die methodische Qualität veröffentlichter Studien bewertet werden.

**Methoden:**

In 3 elektronischen Datenbanken, PubMed, Embase und Cochrane Library, wurde nach relevanten, bis zum 2. März 2024 veröffentlichten Studien gesucht. Die Artikel wurden von 2 Prüfern unabhängig voneinander anhand von Ein- und Ausschlusskriterien gesichtet. Eine Metaanalyse wurde mit der Software RevMan 5.3 unter Verwendung eines Random-Effects-Modells durchgeführt.

**Ergebnisse:**

Insgesamt 18 Studien (Interventionsgruppe: *n* = 1794, Kontrollgruppe: *n* = 2129) wurden in die systematische Überprüfung einbezogen, 18 Studien in die Metaanalyse. Eine frühzeitige Mobilisierung, so das Ergebnis, senkt das Delir-Risiko bei kritisch kranken Patienten, mit einem gepoolten Odds Ratio von 0,65 (95%-Konfidenzintervall [KI]: 0,49–0,86; *p*  = 0,003; I^2^ = 59%). Darüber hinaus ergaben 2 Studien, dass eine frühzeitige Mobilisierung die Dauer des Delirs kritisch kranker Patienten nicht veränderte, gepoolte mittlere Differenz −1,53 (95% KI −3,48–0,41; *P* = 0,12; I^2^ = 37%). Die Analyse der Untergruppen ergab, dass die Frühmobilisierung ihre vorbeugende Wirkung auf Delir in den Vorher‑/Nachher-Interventionsstudien, in vor 2018 veröffentlichten Studien und in Studien, deren methodisch Qualität als mäßig bewertet wurde, beibehielt.

**Schlussfolgerung:**

Als eine nichtpharmakologische Intervention kann die Frühmobilisierung dazu beitragen, das Delir-Risiko zu senken und die Dauer des Delirs bei kritisch kranken Patienten im Vergleich zur Standardbehandlung zu verkürzen. Sie könnte in Zukunft eine innovative Strategie für die Delir-Prävention in der Intensivtherapie darstellen.

## Introduction

Delirium is a common complication in critically ill patients, with a systematic review of studies from North and South America, Europe and Asia reporting a pooled prevalence of 31.8% in patients receiving ventilated and nonventilated treatment in the intensive care unit (ICU). The prevalence of delirium is generally 50–70% in patients receiving mechanically ventilated care, and, accordingly, is of considerable clinical importance [1–4]. Delirium refers to a clinical state that is characterised by a combination of features defined by diagnostic systems such as the DSM‑5 [5]. In critically ill patients, delirium typically manifests as fluctuations in consciousness, abnormal behaviour and cognitive impairment [6]. Although the exact mechanism of delirium remains unclear, research shows that it may be related to many factors, including inflammatory reactions, neurotransmitter imbalance, brain injury and drug use. Additionally, the brain network theory suggests that delirium is associated with disrupted functional connectivity and network efficiency in the brain. This theory posits that hypoactive delirium, in particular, involves changes in brain network dynamics, which can be observed through functional connectivity and network analysis [7]. In critically ill patients in particular, prolonged bed rest, systemic inflammatory responses caused by illness and the use of sedative medications may aggravate the occurrence of delirium, which can be relieved through drug and nondrug intervention [8, 9]. Delirium can have catastrophic clinical consequences for patients [10]. First, it significantly increases the length of hospitalisation time and medical expenses [11]. Second, it leads to failed weaning from mechanical ventilation, muscle atrophy and malnutrition, thereby increasing the risk of ventilator-associated pneumonia and deep vein thrombosis. Additionally, the condition causes anxiety and depression and can even increase the risk of long-term cognitive impairment, severely affecting the quality of life and rehabilitation process of patients [12].

In addition to traditional pharmacological treatments, nonpharmacological interventions for preventing delirium offer a promising approach. Among these nonpharmacological interventions, early mobilisation may be an effective approach. Early mobilisation aims to promote patients’ activity and cognitive function, reduce bed rest time and improve muscle strength and endurance, thereby enhancing their rehabilitation process [13]. The definition of early mobilisation encompasses a range of activities, including in-bed exercises, bedside sitting, walking training and stationary cycling [14]. These activities are provided to patients individually or as part of a bundle. The preventive effect of early mobilisation on delirium is worth exploring. Studies have suggested that early mobilisation can reduce the risk of delirium in critically ill patients [15, 16]. By reducing bed rest time and improving muscle strength and cognitive function, early mobilisation helps to maintain patients’ clarity of consciousness and perception, thereby reducing the incidence of delirium [17]. Additionally, early mobilisation can improve patients’ psychological status and reduce the occurrence of anxiety and depression, further lowering the risk of delirium. However, the effects of early mobilisation on delirium prevention are inconsistent. Although some studies support the effectiveness of this approach, others have failed to confirm its preventive effect on delirium [18]. This can be attributed to differences in study design, sample size and intervention measures.

Although a few meta-analyses have explored the preventive effects of early rehabilitation on delirium in critically ill patients, they include limitations [19, 20]. These meta-analyses are often influenced by factors such as a limited search scope, an insufficient number of included studies and inconsistent methodological quality, leading to ineffective coverage and comprehensive analysis of all the relevant studies in this field. Additionally, because of the rapid development in medical research, new studies continue to emerge, necessitating an up-to-date meta-analysis on the effects of early mobilisation on delirium prevention in critically ill patients. This study aims to address this research gap by exploring whether early mobilisation, either alone or as part of a preventive bundle, can improve the risk or duration of delirium in critically ill patients compared with standard treatment.

## Methods

This meta-analysis was conducted following the Preferred Reporting Items for Systematic Reviews and Meta-Analyses guidelines recommended by the Cochrane Collaboration [21].

### Search strategy and study selection

A systematic search was performed using three electronic databases, PubMed, Embase and the Cochrane Library, from inception to 2 March 2024, without language restrictions. The search strategy combined MeSH terms or Emtree indexing with free-text terms, primarily including ‘delirium’, ‘rehabilitation’, ‘exercise’, ‘early mobilisation’, ‘critical illness’ and ‘ICU’. Free-text terms were searched for in the title and abstract/keyword fields. Details of the search strategy are presented in Table 1. Additionally, the reference lists of relevant studies and reviews were manually searched to identify additional potential records. The retrieved records were imported into EndNote X9.3.3 (Clarivate Analytics, London, UK) for duplicate removal using software and manual screening thereafter. Subsequently, the titles and abstracts of potentially relevant electronic records were screened independently by two reviewers to create a list for full-text reading. Finally, studies that met the inclusion and exclusion criteria were determined according to predefined criteria. When discrepancies occurred between the two reviewers, a senior investigator made the final decision.

**Table 1:** Search strategy for electronic databases

Databases	Search terms
PubMed (*n* = 544)	(“Delirium”[Title/Abstract] OR “confusion”[Title/Abstract] OR “icu psychosis”[Title/Abstract] OR “postoperative psychosis”[Title/Abstract] OR “acute encephalopathy”[Title/Abstract] OR “agitation”[Title/Abstract] OR “icu delirium”[Title/Abstract] OR “Delirium”[MeSH Terms]) AND (“Rehabilitation”[MeSH Terms] OR “Exercise”[MeSH Terms] OR “mobilization”[Title/Abstract] OR “early mobilization”[Title/Abstract] OR “Rehabilitation”[Title/Abstract] OR “Exercise”[Title/Abstract] OR “ambulation”[Title/Abstract] OR “walking”[Title/Abstract] OR “cycling”[Title/Abstract]) AND (“Critical Care”[MeSH Terms] OR “Critical Illness”[MeSH Terms] OR “Intensive Care Units”[MeSH Terms] OR “Critical Illness”[Title/Abstract] OR “Critical Care”[Title/Abstract] OR “intensive care unit”[Title/Abstract] OR “ICU”[Title/Abstract])
Embase (*n* = 1148)	(‘delirium’/exp OR ‘delirium’:ab,ti OR ‘confusion’:ab,ti OR ‘icu psychosis’:ab,ti OR ‘postoperative psychosis’:ab,ti OR ‘acute encephalopathy’:ab,ti OR ‘agitation’:ab,ti OR ‘icu delirium’:ab,ti) AND (‘mobilization’/exp OR ‘rehabilitation’/exp OR ‘exercise’/exp OR ‘mobilization′:ab,ti OR ‘early mobilziation’:ab,ti OR ‘walking’:ab,ti OR ‘cycling’:ab,ti OR ‘ambulation’:ab,ti) AND ((‘critical care’/exp OR ‘critical illness’/exp) AND ‘intensive care unit’/exp OR ‘intensive care unit’:ab,ti OR ‘critical illness’:ab,ti OR ‘critical care’:ab,ti OR ‘icu’:ab,ti)
Cochrane Library (*n* = 252)	((MeSH descriptor: [Delirium] explode all trees) OR (delirium OR confusion OR icu psychosis OR postoperative psychosis OR acute encephalopathy OR agitation OR icu delirium):ti,ab,kw) AND ((MeSH descriptor: [Rehabilitation] explode all trees) OR (MeSH descriptor: [Exercise] explode all trees) OR (mobilization OR early mobilization OR rehabilitation OR exercise OR ambulation OR walking OR cycling):ti,ab,kw) AND ((MeSH descriptor: [Critical Care] explode all trees) OR (MeSH descriptor: [Critical Illness] explode all trees) OR (MeSH descriptor: [Intensive Care Units] explode all trees) OR (critical care OR critical illness OR intensive care unit OR ICU):ti,ab,kw)

### Inclusion and exclusion criteria

Based on the widely applied PICOS (patient, intervention, comparison, outcome, and study design) principle, the inclusion criteria for this study were set as follows: 1) critically ill patients or those admitted to the ICU; 2) intervention groups receiving early mobilisation intervention; 3) the presence of a control group receiving positive or negative control treatments; 4) treatment outcome indicators including delirium incidence or duration; and 5) study designs comprising randomised controlled trials (RCTs), before/after intervention studies (BAISs) or quality improvement studies. Exclusion criteria included the following: 1) duplicate studies; 2) irrelevant study types, such as case reports, literature reviews and conference abstracts; and 3) studies with incomplete data or unclear assessment methods.

The term ‘delirium’ refers to a clinical state characterised by a combination of features that are defined by diagnostic systems such as the DSM‑5. Delirium, according to the DSM‑5, is defined if criteria A–E are fulfilled [5]: A) a disturbance in attention (i.e. a reduced ability to direct, focus, sustain and shift attention) and awareness (reduced orientation to the environment); B) the disturbance, which develops over a short period of time (usually hours to a few days), represents a change from baseline attention and awareness, and tends to fluctuate in severity during the course of the day; C) an additional disturbance in cognition (e.g. memory deficit, disorientation, language, visuospatial ability or perception); D) the disturbances in criteria A and C are not explained by another pre-existing, established or evolving neurocognitive disorder and do not occur in the context of a severely reduced level of arousal, such as a coma; E) there is evidence from the patient’s history, physical examination or laboratory findings that the disturbance is a direct physiological consequence of another medical condition, substance intoxication or withdrawal (e.g. because of a drug abuse or medication) or exposure to a toxin, or is the result of multiple aetiologies. This is a preferred term.

### Data extraction and risk-of-bias assessment

Upon finalising the included studies, two reviewers independently extracted data from the included studies using a pre-designed form. The extracted data mainly included the study author, publication year, country, study design, sample size, intervention and control group settings, ICU type, mechanical ventilation settings, delirium assessment methods, personnel and characteristics of the included population. Additionally, a bias risk assessment of RCTs was conducted following the *Cochrane Handbook for Systematic Reviews of Interventions* (version 5.1) and evaluated aspects such as random sequence generation (selection bias), allocation concealment (selection bias), blinding of participants and personnel (performance bias), blinding of the outcome assessment (detection bias), completeness of the outcome data (attrition bias), selective reporting (reporting bias) and other sources of bias. The risk-of-bias assessment for non-RCTs was conducted using the ROBINS‑I tool [22], which has 7 domains: bias due to confounding, bias in the selection of participants for the study, bias in the classification of interventions, bias due to deviations from intended interventions, bias due to missing data, bias in the measurement of outcomes and bias in the selection of the reported result. Each domain is graded as low, moderate, serious or critical.

### Statistical analysis

Statistical analysis was conducted using RevMan 5.3 software (Nordic Cochrane Centre, Cochrane Collaboration, Copenhagen, Denmark). For dichotomous variables, odds ratios (ORs) with their 95% confidence intervals (CIs), were calculated as the effect-size statistic. For continuous variables, mean differences (MDs) were used. Heterogeneity among the included studies was assessed using the Cochran Q test combined with the I^2^ statistic. When heterogeneity was low (*P* > 0.1 or I^2^ < 50%), a fixed-effects model was employed for meta-analysis; otherwise, a random-effects model was used. The significance level was set at α = 0.05, and publication bias was assessed using funnel plots. Subgroup analyses were conducted based on the study design, publication year and risk of bias to evaluate the effect of early mobilisation on delirium under different conditions. Sensitivity analyses were also performed to evaluate the influence of individual studies on the summary results.

## Results

**Fig. 1:**
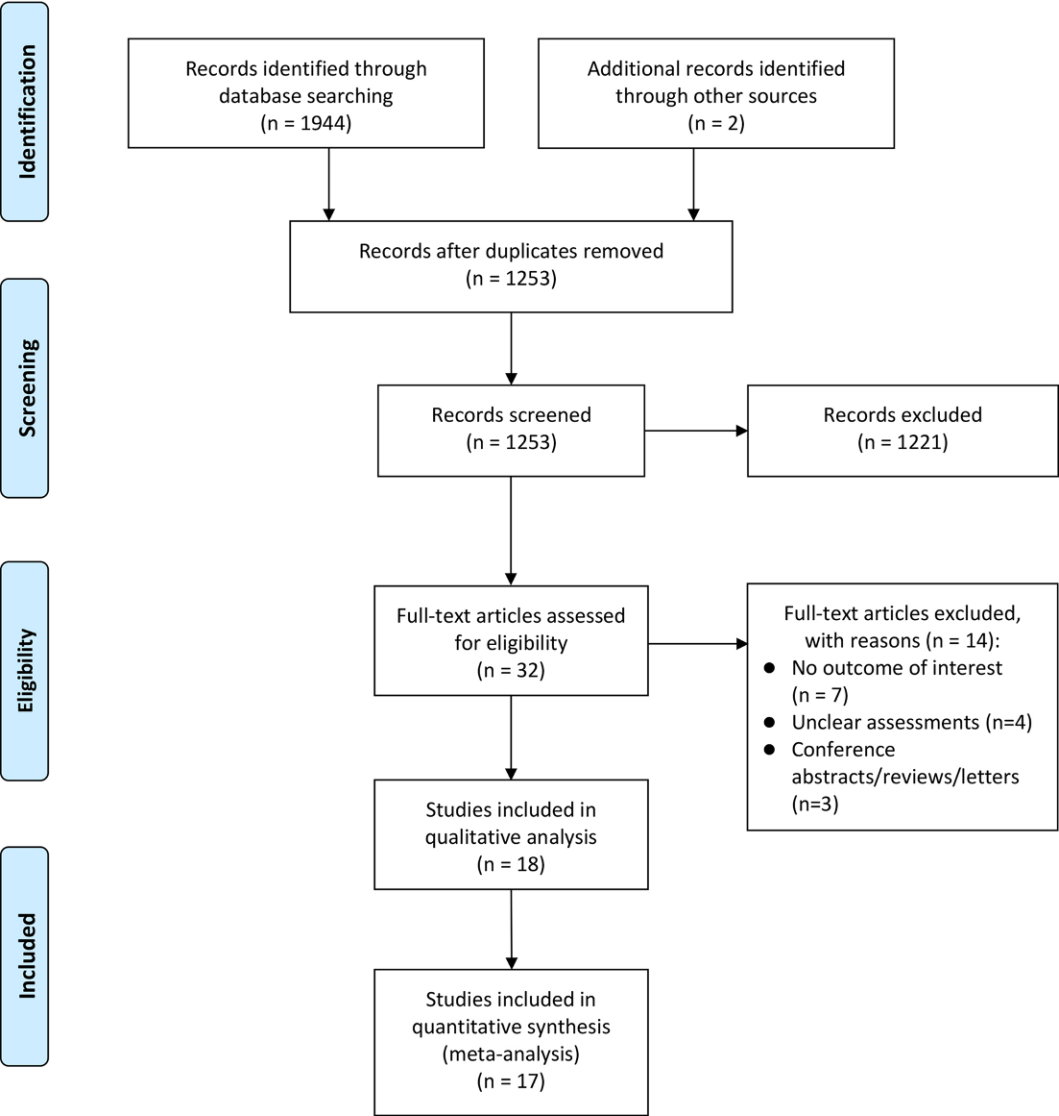
Flow diagram of study screening and selection

Based on the search strategy, a total of 1944 electronic records were obtained, with 544 from PubMed, 1148 from Embase, 252 from the Cochrane Library and an additional 2 records retrieved through other resources. After removing 693 duplicate records, the remaining 1253 records were screened based on titles and abstracts. Full-text reading was conducted for 32 potentially relevant articles, resulting in the exclusion of 14 articles because of unreported outcome settings (*n* = 7), unclear assessment (*n* = 4) and irrelevant study types (*n* = 3). Finally, 18 articles were included in the systematic review, 1 of which was excluded from the meta-analysis because of unavailable data. The detailed literature screening process is shown in Fig. 1.

### Characteristics of the included studies

A total of 18 articles [18, 23–39] were included in the systematic review, and their basic characteristics are summarised in Table 2. Among them, 7 studies were conducted in North America, and there were 7 RCTs, 10 BAISs and 1 quality improvement study. The intervention group comprised 1794 participants, whereas the control group included 2129 participants. Various intervention methods were used in the included studies, with 4 studies adopting the ABCDE bundle method and 14 adopting conventional control methods (Supplementary Table S1). Thirteen studies reported patients mainly from mixed ICUs, whereas the remaining studies reported patients from medical, trauma, neurological and emergency ICUs. Various delirium assessment methods were employed, including the confusion assessment method (CAM), DRS, and ICDSC scores, with the CAM-ICU being the most frequently used (66.7%).

**Table 2:** Main characteristics of included studies

First author (year)	Country	Design	Sample size (intervention vs. control)	Intervention	Control	Type of ICU	Mechanical ventilation	Delirium assessment	Guidance
Álvarez (2017) [23]	Chile	Single-centre RCT	70 vs. 70	Prevention bundle + occupational therapy	Prevention bundle	Mixed ICU	Without	CAM, DRS	Occupational therapist
Balas (2014) [24]	USA	Single-centre BAIS	146 vs. 150	ABCDE bundle	Usual care	Mixed ICU	Without	CAM-ICU	Mixed Team
Berney (2021) [18]	Australia, USA	Multi-centre RCT	80 vs. 82	Usual rehabilitation + FES-cycling	Usual rehabilitation	Mixed ICU	≥ 48 h	CAM-ICU	Mixed Team
Bounds (2016) [25]	USA	Single-centre BAIS	80 vs. 79	ABCDE bundle	Usual care	Mixed ICU	≥ 24 h	ICDSC	Mixed Team
Bryant (2019) [26]	USA	Single-centre BAIS	125 vs. 144	The Frailty Identification and Care Pathway	Usual care	Trauma ICU	Without	CAM	Interdisciplinary team
Choong (2024) [27]	Canada	Single-centre BAIS	293 vs. 200	Early rehabilitation bundle	Usual care	Mixed paediatric ICU	Without	CADPS	Mixed Team
Foster (2013) [28]	USA	Single-centre BAIS	92 vs. 164	Prevention bundle	Usual care	Medical ICU	Without	CAM-ICU	Registered nurse
Frade-Mera (2022) [29]	Spain	Multi-centre BAIS	51 vs. 554	Part of ABCDE bundle	ABCDE bundle without early mobilisation	Mixed ICU	≥ 48 h	CAM-ICU	Physiotherapist
Karadas (2016) [30]	Turkey	Single-centre RCT	47 vs. 47	Assisted-active range of exercises	Usual care	Adult medical ICU	Noninvasive	CAM-ICU	NR
Larsen (2020) [31]	Denmark	Single-centre BAIS	50 vs. 39	Prevention bundle	Usual care	Neurological ICU	Without	ICDSC	NR
Lee (2019) [32]	Korea	Single-centre BAIS	94 vs. 91	Modified ABCDE bundle	Early ABCDE bundle	Mixed ICU	Without	CAM-ICU	Mixed Team
Martínez (2017) [33]	Chile	Single-centre BAIS	227 vs. 60	Prevention bundle	Usual care	Mixed ICU	Without	CAM-ICU	Physiotherapist
Matsuki (2020) [34]	Japan	Single-centre QI project	37 vs. 18	Rehabilitation protocol + physiotherapist	Usual care	Emergency ICU	Without	ICDSC	Physiotherapist
Moon (2015) [35]	Korea	Single-centre RCT	60 vs. 63	Prevention bundle	Usual care	Mixed ICU	Without	CAM-ICU	Mixed Team
Nydahl (2020) [36]	Germany	Multi-centre RCT	120 vs. 152	Out-of-bed mobilisation	Usual care	Mixed ICU	Without	CAM-ICU, ICDSC	Mixed Team
Nydahl (2021) [37]	Germany, UK	Multi-centre RCT	26 vs. 20	Out-of-bed mobilisation	Usual care	Mixed ICU	Without	CAM-ICU	Mixed Team
Patel (2014) [38]	UK	Single-centre BAIS	171 vs. 167	Prevention bundle	Usual care	Mixed ICU	Without	CAM-ICU	Registered nurse
Winkelman (2018) [39]	USA	Multi-centre RCT	25 vs. 29	Twice daily mobilisation	Once daily mobilisation	Mixed ICU	≥ 36 h	CAM-ICU	Registered nurse

*ICU* intensive care unit, *RCT* randomized controlled trial, *CAM* Confusion Assessment Method, *DRS* Delirium Rating Scale, *USA* United States of America, *BAIS* before-after intervention study, *ABCDE* Awakening and Breathing Coordination, Delirium monitoring/management, and Early exercise/mobility, *FES* functional electrical stimulation, *ICDSC* Intensive Care Delirium Screening Checklist, *CADPS* Cornell Assessment for Delirium in Pediatrics Score, *QI* quality improvement

The characteristics of patients included in the studies are presented in Table 3. The mean age ranged from 34.3–82.9 years in the intervention group and from 28.3–84.3 years in the control group. The proportion of men ranged from 41.7–73.1% and from 36.8–70.7%, respectively. The APACHE II scores ranged from 10–26.6 and from 11–26.3, respectively, and the delirium rates ranged from 0–95% and from 0–92.8%, respectively.

**Table 3:** Patient characteristics in included studies

First author (year)	Intervention					Control				
	Age, years	Men, %	APACHE II	Mechanical ventilation, %	Delirium incidence, %	Age, years	Men, %	APACHE II	Mechanical ventilation, %	Delirium incidence, %
Álvarez (2017) [23]	68 (63–75.5)	50.6	10 (9–12)	0	3	71 (63–78.5)	41.6	11 (8–12)	0	20
Balas (2014) [24]	55.6 ± 14.9	57.3	21 (16–28)	62.7	49	59.2 ± 16.1	54.1	23.5 (17–29)	63.7	62
Berney (2021) [18]	61 (51–69)	66	22 (16–27)	100	59	59 (48–67)	66	23 (17–27)	100	55
Bounds (2016) [25]	65.3 ± 15.5	62	NR	39.2	23	67.2 ± 14.6	55	NR	41.2	38
Bryant (2019) [26]	82.9 ± 7.4	41.7	NR	NR	12.5	84.3 ± 6.7	36.8	NR	NR	21.6
Choong (2024) [27]	34.3 (5.1–115.5); 74.8 (12.2–132.4)	53.0; 63.6	NR	58; 61	20.1	28.3 (6.1–127.0); 36.3 (4.0–96.4)	57.3; 54.1	NR	47; 89	22.5
Foster (2013) [28]	NR	NR	NR	NR	28	NR	NR	NR	NR	28
Frade-Mera (2022) [29]	67 (53–77)	60.8	24 (19–28)	NR	0	65 (54–74)	70.7	21 (26–27)	NR	0
Karadas (2016) [30]	75 ± 7.5	51.1	NR	NR	8.5	72.6 ± 6.8	42.6	NR	NR	21.3
Larsen (2020) [31]	62 (50–70)	54	20.5 ± 7.2	70	88	62 (52–72)	46	22.6 ± 6.9	69	90
Lee (2019) [32]	64.8 ± 16.6	55.3	26.6 ± 7.6	38.3	75.5	64.4 ± 13.9	59.3	26.3 ± 7.7	30.8	67.0
Martínez (2017) [33]	63.5 ± 18.4	55	NR	51.1	38	62.8 ± 17.8	55	NR	50	24
Matsuki (2020) [34]	71.2 ± 12.5	51.3	19.1 ± 7.7	51	38	78.4 ± 6.1	50	19.8 ± 8.7	50	53
Moon (2015) [35]	70 ± 13.8	50	13 ± 5.8	16.7	20	69 ± 12.4	46	14.9 ± 6.2	25.3	33
Nydahl (2020) [36]	74 (61–81)	54.1	NR	34.8	95	70 (58–79.7)	53.3	NR	31.1	92.8
Nydahl (2021) [37]	64.4 ± 11.9	73.1	NR	69.2	26.9	60 ± 17.3	70	NR	50	50
Patel (2014) [38]	62.8 ± 17.8	53	14.2 ± 6.6	NR	14	63.5 ± 18.4	51	15.0 ± 7.6	NR	33
Winkelman (2018) [39]	52.7 ± 18.5	56	NR	100	47.1	59.5 ± 15.6	38	NR	100	52.4

*APACHE II* Acute Physiology and Chronic Health Evaluation II, *NR* not reported

### Risk-of-bias assessment

**Fig. 2:**
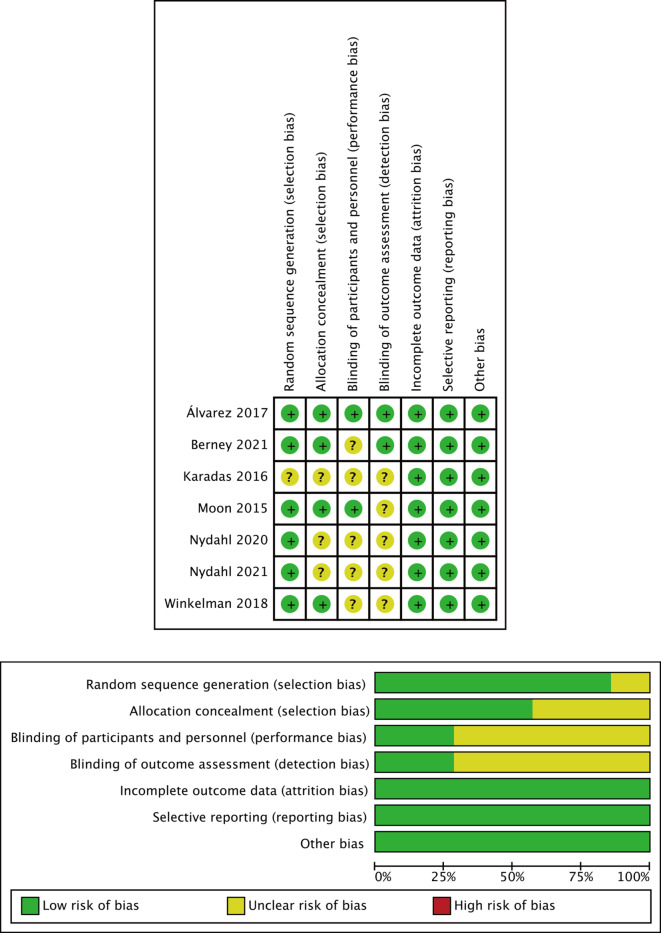
Methodological quality of randomized controlled trials assessed with the Cochrane Risk of Bias tool

In terms of RCTs, detailed information regarding the risk of bias is shown in Fig. 2. The majority of studies (85.7%) clearly reported methods for random sequence generation, although some studies reflected an unclear risk, caused by allocation concealment and blinding factors. No RCT had incomplete outcome data, obvious selective reporting or other biases. Regarding non-RCTs, detailed information about the risk of bias is shown in Table 4. Most studies (81.8%) were rated as moderate, but 1 study was rated as serious and 1 as critical.

**Table 4:** Methodological quality of nonrandomized controlled trials assessed with the ROBINS‑I tool

First author (year)	Confounding	Selection bias	Classification of interventions	Deviations from interventions	Missing data	Measuring outcomes	Reporting bias	Overall
Balas (2014) [24]	Moderate	Low	Low	Low	Low	Low	Low	Moderate
Bounds (2016) [25]	Low	Low	Low	Low	Low	Moderate	Low	Moderate
Bryant (2019) [26]	Serious	Low	Low	Low	Low	Moderate	Low	Serious
Choong (2024) [27]	Moderate	Low	Low	Low	Low	Moderate	Low	Moderate
Foster (2013) [28]	Critical	Low	Serious	Low	Moderate	Moderate	Low	Critical
Frade-Mera (2022) [29]	Low	Low	Moderate	Low	Low	Low	Low	Moderate
Larsen (2020) [31]	Moderate	Low	Low	Low	Moderate	Moderate	Low	Moderate
Lee (2019) [32]	Moderate	Low	Moderate	Low	Low	Low	Low	Moderate
Martínez (2017) [33]	Moderate	Low	Low	Low	Low	Moderate	Low	Moderate
Matsuki (2020) [34]	Low	Low	Moderate	Low	Low	Low	Low	Moderate
Patel (2014) [38]	Moderate	Low	Low	Low	Low	Moderate	Low	Moderate

*ROBINS‑I* risk of bias in nonrandomized studies of interventions tool

### Meta-analysis

Data on delirium incidence were reported in 17 studies. After pooling the data, it was found that early mobilisation reduced the risk of delirium in critically ill patients, with a summary OR of 0.65 (95% CI 0.49–0.86; *P* = 0.003; I^2^ = 59%). Additionally, data on delirium duration were reported in 2 studies, revealing that early mobilisation did not change the duration of delirium in critically ill patients, with a summary MD of −1.53 (95% CI: −3.48–0.41; *P* = 0.12; I^2^ = 37%).

### Subgroup analysis

**Fig. 3:**
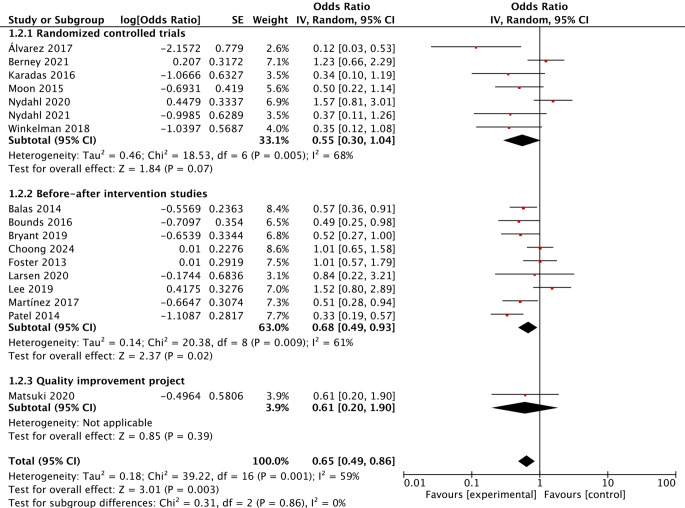
Forest plot of the effect of early mobilisation on prevention of delirium in critically ill patients stratified by study design

**Fig. 4:**
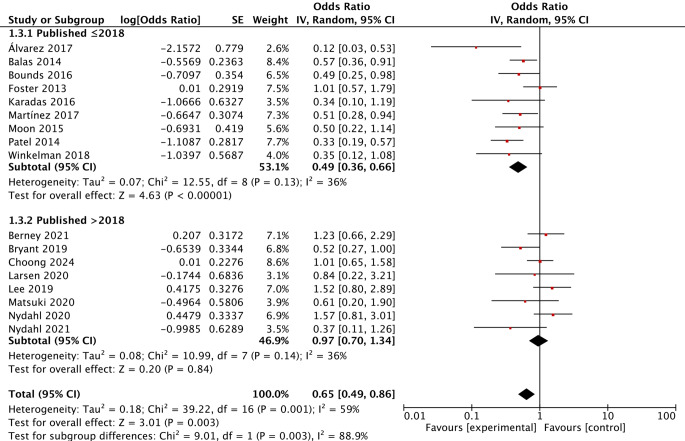
Forest plot of the effect of early mobilisation on prevention of delirium in critically ill patients stratified by published years

**Fig. 5:**
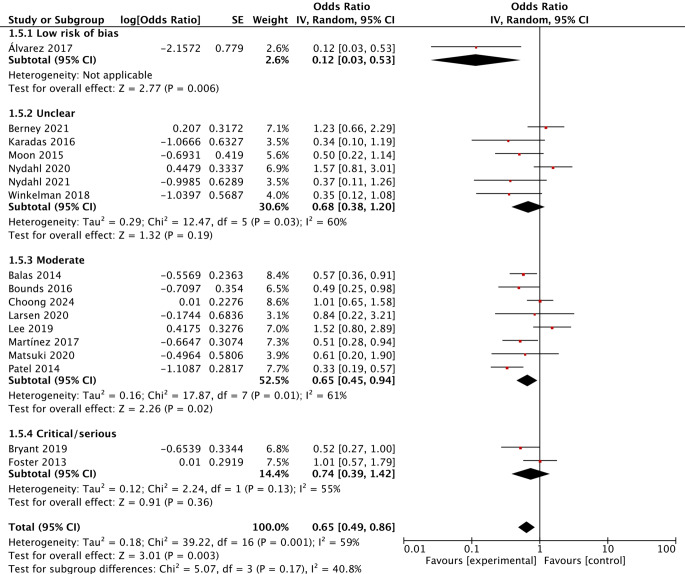
Forest plot of the effect of early mobilisation on prevention of delirium in critically ill patients stratified by risk of bias

Subgroup analyses were conducted based on study design, publication year and risk of bias to evaluate the effect of early mobilisation on delirium. The results showed that early mobilisation did not reduce the risk of delirium in the RCTs, although it effectively prevented delirium in the BAISs, with summary ORs of 0.55 (95% CI 0.30–1.04; *P* = 0.07; I^2^ = 68%) and 0.68 (95% CI 0.49–0.93; *P* = 0.02; I^2^ = 61%), respectively. Furthermore, only one quality-improvement study reported a nonsignificant effect of early mobilisation on delirium (Fig. 3). The results of articles published before 2018 showed that early mobilisation effectively reduced the risk of delirium, but this effect was not significant in articles published after 2018, with summary ORs of 0.49 (95% CI 0.36–0.66; *P* < 0.001; I^2^ = 36%) and 0.97 (95% CI 0.70–1.34; *P* = 0.84; I^2^ = 36%), respectively (Fig. 4). According to the risk assessment results, early mobilisation significantly prevented delirium only in studies rated as moderate, with a summary OR of 0.65 (95% CI 0.45–0.94; *P* = 0.02; I^2^ = 61%; Fig. 5).

### Publication bias and sensitivity analysis

**Fig. 6:**
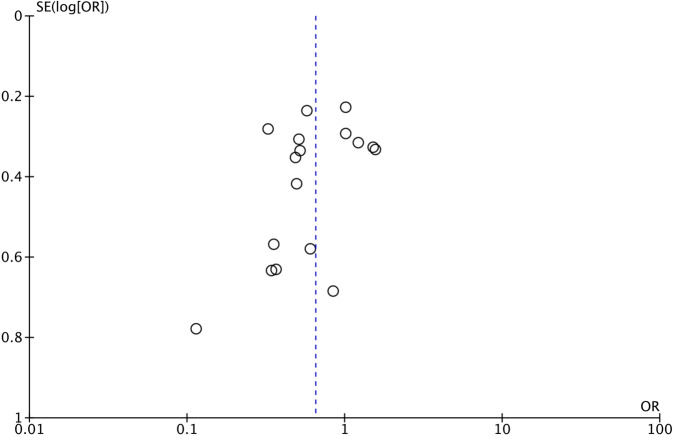
Funnel plot of the effect of early mobilisation on prevention of delirium in critically ill patients. *SE* standard error, *OR* odds ratio

After conducting funnel plot analysis for delirium incidence data, it was found that the distribution of results from 17 studies was approximately symmetrical, indicating no significant publication bias (Fig. 6). Furthermore, excluding any single study did not significantly alter the summary results, suggesting a limited influence of individual studies on the overall results.

## Discussion

This meta-analysis synthesised the latest clinical evidence to evaluate the preventive and therapeutic effects of early mobilisation as a nonpharmacological intervention for delirium in critically ill patients and assessed the risk of bias in the included studies. The findings support the brain network theory, which suggests that interventions improving brain connectivity, such as early mobilisation, may reduce the incidence of delirium. Studies utilising encephalography (EEG) have highlighted the importance of maintaining functional connectivity to prevent delirium. Furthermore, Schaller et al. [40] emphasised that early mobilisation should be initiated within the first 72 h of ICU admission to optimise outcomes and minimise the risk of complications. The main findings of this study are as follows: 1) compared with the control group, critically ill patients who received early mobilisation had a significantly reduced risk of delirium, but the treatment did not reduce the duration of the condition; 2) early mobilisation effectively prevented delirium in BAISs but did not reduce the risk of delirium in RCTs; and 3) publication year and study quality partially influenced the effect of early mobilisation on preventing delirium. This study is anticipated to provide evidence-based guidelines for the clinical use of early mobilisation to prevent delirium in critically ill patients and serve as a reference for future research in this area.

The results of this study indicate that the majority of studies support early mobilisation as an intervention to reduce the risk of delirium in critically ill patients. This aligns with the brain network theory, which emphasises the role of functional connectivity in maintaining cognitive function and reducing delirium risk. Early mobilisation may enhance brain network efficiency and connectivity, contributing to its preventive effects on delirium [41]. Additionally, the findings support the updated hypothesis by Maldonado et al. [42], which suggests that addressing neurotransmitter dysregulation, inflammation and neuroendocrine dysregulation can mitigate delirium risk in critically ill patients. Furthermore, according to Schaller et al. [40], early mobilisation protocols, when implemented within the first 72 h of ICU admission, can significantly improve patient outcomes. Additionally, a study conducted by Nydahl et al. [43] highlights the global implementation of delirium assessment protocols and the importance of standardised approaches, including early mobilisation as part of a bundled intervention, to effectively manage and prevent delirium in critically ill patients. However, to translate these findings into clinical practice, it is crucial to consider how early mobilisation can be effectively integrated into standard ICU care protocols. The study results suggest that early mobilisation, particularly when initiated early, can have significant impacts on patient outcomes, such as reducing the incidence of delirium, improving cognitive function, and potentially shortening ICU stays. The evidence points toward early mobilisation as a key nonpharmacological strategy for delirium prevention, which could be incorporated into routine ICU protocols as part of a holistic approach to patient care.

Despite these promising findings, inconsistencies remain across studies. Differences in study design, sample size, and intervention methods, as well as variations in brain connectivity measures, may contribute to these discrepancies. Moreover, the timing and intensity of early mobilisation interventions, as highlighted by Schaller et al. [40], may influence the outcomes and effectiveness of the intervention. In clinical practice, implementing early mobilisation as a standalone intervention may not always be sufficient, particularly in patients with severe shock, infection, or other complicating factors. In these cases, early mobilisation should be considered part of a broader, multifaceted care bundle that addresses the complex pathophysiology of delirium.

This analysis underscores the importance of early mobilisation in stroke patients, though its effects and optimal timing require further investigation. For instance, a study on very early mobilisation after thrombolysis in acute ischemic stroke patients found no significant improvement in functional outcomes or secondary outcomes within the first 24 h [44]. Similarly, the AVERT trial, which assessed very early mobilisation within 24 h, found no improvement in quality of life at 3 or 12 months compared to usual care. In fact, this study suggested that mobilisation initiated too early might even have detrimental effects, particularly in the short term [45, 46]. Current evidence generally supports starting mobilisation after 24 h, once patients have achieved haemodynamic stability, as this timing is associated with improved functional recovery [47].

The study by Nydahl et al. [43] highlights the need for global standardisation in delirium assessment and management protocols to address inconsistencies in intervention effectiveness. Early rehabilitation, especially early mobilisation, is an essential part of delirium prevention in critically ill patients, yet its effectiveness may vary depending on patient characteristics and the clinical environment. Several studies suggest that early mobilisation, when incorporated into multicomponent intervention bundles, can reduce delirium incidence and duration. For instance, the ABCDEF bundle, which includes early mobility as a key component, has been shown to decrease delirium and ICU stay duration in ICU patients [48]. Additionally, nonpharmacological interventions like exercise and cognitive stimulation, part of structured rehabilitation programs, have demonstrated efficacy in reducing delirium [49]. However, as delirium can be caused by various factors—such as shock, infection and hypoxia—it is crucial to consider a comprehensive approach rather than relying solely on early mobilisation. In cases like stroke patients, reduced movement can lead to poorer cerebral perfusion, increasing the risk of delirium [50]. On the other hand, premature mobilisation in critical conditions like shock might exacerbate hypoperfusion and worsen delirium risk [51]. Network meta-analysis further reinforces the role of multicomponent interventions, including early mobilisation, in delirium prevention, with some combinations like sleep promotion, cognitive stimulation, and early mobilisation proving particularly effective [20]. Therefore, it is essential that early rehabilitation and mobilisation programs be tailored to individual patient needs, ensuring personalised care that considers their specific clinical circumstances.

The results of this study are consistent with previous meta-analyses [19, 20], indicating that early rehabilitation may have a significant effect in preventing delirium in critically ill patients. However, this study offers several novel contributions that differentiate it from earlier research. First, we synthesised the latest research evidence, incorporating studies published up to 2024, which include findings that previous meta-analyses may have missed. By expanding the scope of the evidence base, our analysis provides a more comprehensive and up-to-date confirmation of early mobilisation’s effect on delirium prevention. This ensures that our conclusions are reflective of the most current research, reinforcing the importance of early mobilisation as a key intervention for delirium. Second, our meta-analysis features a more thorough subgroup analysis, allowing for a deeper understanding of the effect of early mobilisation across different clinical scenarios. We explored variations in patient characteristics, study design, and intervention timing, which helped explain the heterogeneity observed in the overall results. This level of granularity offers more nuanced insights into the effectiveness of early mobilisation under specific conditions, such as in before/after intervention studies, or studies conducted prior to 2018. By analysing different patient subgroups, we provide a clearer picture of the mechanisms underlying early rehabilitation and its impact on delirium prevention, thereby offering more precise guidance for clinical practice. Third, this study highlights the time-dependent effect of early mobilisation, showing that the intervention’s efficacy may vary based on evolving knowledge and practices in ICU care. This dynamic perspective contributes a fresh understanding of how early mobilisation protocols can be refined and adapted to improve patient outcomes over time. In summary, while this meta-analysis confirms findings from previous research, it adds novel value by incorporating the latest studies, offering detailed subgroup analyses, and providing insights into the evolving application of early mobilisation in ICU settings. These aspects strengthen the evidence base for early rehabilitation and provide more actionable recommendations for its integration into routine clinical practice.

Although the results of this study are largely credible, it still includes limitations. First, although most studies did not have a clear high risk of bias, some uncertainty remains, which may have affected the study results to an extent. Second, although we attempted various methods to explain the heterogeneity in the study, the overall results still exhibited significant heterogeneity. This may have been the result of, for example, differences in study design, measurement methods or population characteristics, and further research is needed to explain this. Moreover, how to influence the results if different subgroup analysis methods are adopted remain requires further investigation. Nonetheless, it remains certain that early exercise can reduce the risk of mental illness in critically ill patients. Finally, we will focus on various clinical interferences or the pathophysiological mechanisms of the disease itself that affect the advantage of early exercise. Overall, our conclusions must be validated by additional studies and should be cautiously generalised to clinical practice.

## Conclusion

Compared with standard care, as a nonpharmacological intervention, early mobilisation can help to reduce the risk of delirium and decrease its duration in critically ill patients. It holds promise as a novel strategy for preventing delirium in the ICU setting. However, considering the heterogeneity observed in this meta-analysis, further high-quality research is needed to explore this topic in more depth.

## Supplementary Information

**Supplementary Table S1. **Details of intervention and control groups

## Data Availability

All data generated or analysed during this study are included in this published article.

## Declarations

Ethical statement is non-applicable for this study due to the design of a meta-analysis.

The supplement containing this article is not sponsored by industry.
