# Supplementary material for: Preventive effects of early mobilisation on delirium incidence in critically ill patients: systematic review and meta-analysis
Source: Med Klin Intensivmed Notfmed. 2025 Mar 14;120(Suppl 1):15–28. doi: 10.1007/s00063-024-01243-8 (PMC12708774; doi:10.1007/s00063-024-01243-8)
Supplement: Supplementary file 1 — Supplementary Table S1. Details of intervention and control groups [file 63_2024_1243_MOESM1_ESM.docx]

**Supplementary Table S1.** Details of intervention and control groups

| **First author, year** | **Control group** | **Intervention group** |
| --- | --- | --- |
| Álvarez, 2017 [23] | Patients received standard non-pharmacological delirium prevention (nPP), including reorientation twice daily, early mobilization three times daily by a physiotherapist, sensory correction (glasses/hearing aids), environmental management (clocks, calendars, reduced restraints), a sleep protocol (dimmed lights, quiet), and avoidance of delirium-inducing medications. | In addition to standard nPP, patients in the intervention group received early and intensive occupational therapy starting within 24 hours of ICU admission. Therapy included two 40-minute sessions daily focusing on polysensory stimulation, cognitive exercises, upper extremity motor function, basic daily activities, and family participation. |
| Balas, 2014 [24] | Clinicians inconsistently performed spontaneous awakening trials (SATs) and spontaneous breathing trials (SBTs) without formal policies. No coordinated SAT/SBT procedures, delirium monitoring, or management protocols were in place. Early mobility was limited, with few patients assisted out of bed. | The ABCDE bundle was applied to all ICU patients daily unless opted out by a prescriber. The bundle integrated SATs, SBTs, delirium monitoring, early mobility, and interprofessional rounding with safety and success criteria. |
| Berney, 2021 [18] | The control group received usual care rehabilitation provided by experienced critical care physiotherapists, following local practices. Both groups continued rehabilitation after ICU discharge, and physiotherapists documented active time spent on FES-cycling and usual care rehabilitation. | Participants received FES-cycling with synchronized stimulation of four muscle groups for up to 60 minutes per day, at least 5 days per week. One leg was randomized to FES-cycling, and the other to cycling without FES. This was in addition to usual care rehabilitation as per local practices. |
| Bounds, 2016 [25] | The control group comprised patients who received standard care prior to the ABCDE bundle implementation. Usual care included inconsistent practices around sedation vacations and spontaneous breathing trials, with limited early mobility and no formal delirium monitoring protocols. | Patients aged 18 or older who stayed in the ICU for more than 24 hours were included. Key data collected included demographics, ICU diagnoses, length of stay, days on mechanical ventilation, delirium scores (ICDSC), sedation/awakening trials, and spontaneous breathing trials. The study implemented the ABCDE bundle, focusing on sedation, mobility, and delirium management. |
| Bryant, 2019 [26] | The control group received usual trauma care without the implementation of the standardized Frailty Care Pathway. There was no structured frailty screening or standardized interdisciplinary care protocol. | The Frailty Identification and Care Pathway was developed over six months by an interdisciplinary team to reduce delirium and complications in frail geriatric trauma patients. Patients aged 65+ who screened positive for frailty or cognitive impairment were placed on the pathway, which emphasized early mobilization, nutrition, and delirium prevention. The intervention involved geriatric consultations, physical therapy, and social work within 72 hours of admission. Education for staff included mandatory online training and biweekly rounds. |
| Choong, 2024 [27] | The control group, comprising children admitted before the bundle implementation, received standard PICU care without the structured analgesia, delirium, and mobility interventions. There were no specific guidelines or unit-wide initiatives in place during this period. | The intervention involved a quality improvement bundle consisting of an analgesia-first sedation approach, routine delirium monitoring and prevention, and early mobility-based physical activity. This bundle was implemented across two PICU sites. At Site 1, two interprofessional teams handled evidence-based guideline development and process readiness, while Site 2 had a smaller team managing all implementation activities. Educational resources and progress were shared across sites, with continuous evaluation. |
| Foster, 2013 [28] | Usual care involved variable sedation practices without a structured protocol, limited focus on sleep hygiene, minimal mobility encouragement, and reduced sensory engagement. There was no systematic monitoring or assessment of delirium, resulting in a reactive and less organized approach to managing delirium in critically ill patients. | The multicomponent, nonpharmacologic delirium prevention protocol included daily sedation cessation at 7:30 AM, promotion of sleep-wake cycles with a designated sleep period from 10:00 PM to 4:00 AM, and environmental modifications to reduce noise and distractions. A four-level mobility protocol was implemented based on patient acuity, and patients engaged with sensory aids and preferred music, with monitoring for compliance and effects using validated assessment tools. |
| Frade-Mera, 2022 [29] | Usual care involved a standard approach without structured monitoring or compliance to the ABCDE bundle components. Patients were subjected to variable assessments of pain and delirium management, which potentially led to suboptimal outcomes in pain control, mobility, and sedation practices. The lack of a systematic protocol may have affected overall ICU stay, drug use, and the development of complications such as ICUAW. | The study aimed to evaluate patient outcomes, including pain levels, cooperation, days with delirium, use of physical restraints, mobility, drug levels (analgesics, sedatives, muscle relaxants, antipsychotics), need for re-intubation or tracheostomy, ICU length of stay, days on invasive mechanical ventilation (IMV), bed rest days, ICU mortality, and incidence of ICU-acquired muscle weakness (ICUAW). Compliance with the ABCDE bundle (analgosedation algorithms, delirium prevention, early mobilization) was monitored over a 4-month period in a multicenter cohort study of patients receiving IMV for at least 48 hours. |
| Karadas, 2016 [30] | Patients were monitored during day shifts using RASS and CAM-ICU assessments but received no additional interventions beyond routine clinical practice. This lack of structured exercise may have limited mobility and recovery outcomes compared to the intervention group. | Patients received daily assessments using the Richmond Agitation-Sedation Scale (RASS) and Confusion Assessment Method for the ICU (CAM-ICU). Following these assessments, range of motion (ROM) exercises were conducted once a day until ICU discharge. Depending on patient responsiveness, passive, assisted-active, or active ROM exercises were performed for all four extremities in a supine position, comprising 10 repetitions over approximately 30 minutes. If patients exhibited intolerance (e.g., low blood pressure, high heart rate, low oxygen saturation), exercises were halted and resumed the next day. |
| Larsen, 2020 [31] | Standard care was provided without the structured interventions outlined in the bundle, lacking tailored sedation, enhanced sleep hygiene, optimal pain assessment methods, and systematic mobilization efforts, potentially impacting patient outcomes in the ICU. | Staff received training on the intervention bundle during a one-day workshop, followed by weekly gatherings for reflections and discussions to reinforce study focus. The bundle was based on the best evidence and the 2013 PAD guideline, tailored for local context. Key components included: an analgo-sedation approach to manage pain before sedation, shifting noisy procedures to daytime for improved sleep hygiene, implementing the Critical-Care Pain Observational Tool (CPOT) for pain assessment in non-verbal patients, and promoting early mobilization using a five-level activity hierarchy to encourage patients to achieve the highest possible mobility each day. |
| Lee, 2019 [32] | Introduced the ABCDE bundle for early mobility interventions. Emphasized sedation management, daily assessments, and pain evaluation. Implemented early mobilization based on patient stability. | Enhanced the early ABCDE bundle through continuous quality improvement activities, including education, protocol amendments, and feedback. Modified interventions allowed more patients to participate in early mobility and adjusted screening criteria for exercise therapy. |
| Martínez, 2017 [33] | - Routine clinical practices without the structured interventions outlined in the prevention bundle.  - Standard monitoring and treatment protocols, focusing primarily on patient stabilization and management of critical illness. | - **Physiotherapy and Early Mobilization**: Regular mobilization exercises.  - **Daily Reorientation**: Frequent reorientation of patients.  - **Prevention of Sensory Deprivation**: Ensuring sensory engagement.  - **Avoidance of Delirium-Triggers**: Minimizing use of certain medications.  - **Pain Control**: Effective management of pain.  - **Sleep Hygiene**: Practices to enhance sleep quality.  - **Environmental Stimulation**: Activities to engage patients.  - **Monitoring of Urinary and Rectal Function**: Regular assessments.  - **Minimization of Physical Restraints**: Reducing restraint use.  - **Family Participation**: Involvement of family in care processes. |
| Matsuki, 2020 [34] | - Standard ICU care without a structured rehabilitation protocol (July 2014 - June 2015).  - No specific rehabilitation interventions were implemented. | - Continued use of the rehabilitation protocol with the addition of a dedicated therapist (March 2017 - June 2018).  - Dedicated therapist assigned to the ICU to set protocol levels and oversee interventions.  - Morning interventions led by the dedicated therapist, with additional afternoon interventions by other therapists or nursing staff. |
| Moon, 2015 [35] | - Cognitive function assessed using the CAM-ICU, but no delirium prevention protocol applied.  - Provided typical nursing care without monitoring or screening for delirium risk.  - Care included checking consciousness and orientation but lacked:  1. Reorientation efforts.  2. Nonverbal communication aids.  3. Personal visual or hearing aids.  4. Consistent nurse assignments.  5. Minimization of bed movement.  6. Careful use of medications like anticholinergics and opiates. | - Delirium prevention protocol applied during the first 7 days of ICU hospitalization.  - Small sticker placed at the bedside to indicate group assignment.  - ICU nurses received training (2 sessions of 30 min each) on delirium prevention and management.  - Daily visits by the research team (2 hours) to engage with patients and ensure protocol adherence, including gentle cleaning and safe repositioning. |
| Nydahl, 2020 [36] | Received only standard care, with nurses performing routine checks for consciousness and orientation without implementing the delirium prevention protocol or conducting delirium risk monitoring. Nurses followed standard procedures without additional training for delirium prevention and did not employ extra identification measures. | Implemented a delirium prevention protocol for 7 days, with the research team interacting with patients daily for assessment and care. ICU nurses received training on delirium prevention and management. Delirium was assessed every 12 hours using the CAM-ICU, with personalized care and environmental stimulation to prevent delirium. A small sticker was placed at the bedside to prevent confusion regarding group assignment. |
| Nydahl, 2021 [37] | Patients in the control group received usual care, including mobilization by physiotherapists and nurses during the day, based on clinical judgment. They could also be mobilized in the evening. The mobilization goals were similar to the intervention group, with a focus on sitting, standing, or walking, depending on patient tolerance. The duration of mobilization was also aligned with patient needs, ranging from a minimum of 3 minutes to a maximum of 2 hours. However, they did not have the structured intervention provided to the intervention group. | The intervention involved a dedicated mobilization team consisting of trained ICU nurses and physiotherapists, who provided early mobilization starting from day 1 of ICU admission. Patients were approached for mobilization between 21:00 and 23:00, after informed consent. Mobilization aimed to reach a minimum level of sitting at the edge of the bed, progressing to standing or walking, depending on patient tolerance. The intervention included soothing activities and was carried out for up to three consecutive evenings, adhering to pre-defined safety criteria. If patients were sleeping, in pain, or otherwise unavailable, mobilization was deferred. |
| Patel, 2014 [38] | The control group continued to receive standard care without the structured multicomponent interventions aimed at preventing delirium. Staff did not implement specific measures to reduce noise, light, or iatrogenic sleep disturbances, and no additional training or education sessions were provided regarding delirium prevention strategies. | The intervention was a multidisciplinary multicomponent bundle aimed at reducing factors contributing to delirium. This included measures to minimize noise, light, and iatrogenic sleep disturbances, alongside efforts to modify risk factors for delirium. Staff education and training sessions were conducted multiple times daily to provide background information on sleep and delirium and practical guidance for implementing the interventions. The initiative was further supported by posters displayed in both clinical and non-clinical areas. Eight senior clinical and nursing staff members were designated as 'champions' of the intervention, receiving additional education on delirium prevention and training on the various components of the intervention, serving as a resource for other staff members. |
| Winkelman, 2018 [39] | The control group did not receive the ETM intervention and continued to receive standard care in the ICU without the structured mobilization activities. No specific measures were taken to assess or improve muscle strength or monitor delirium in this group during their ICU stay. | The ETM (Early Therapeutic Mobilization) intervention was delivered by trained registered nurses (RNs) after obtaining informed consent and, when applicable, patient assent. Patients were deemed eligible if they met specific stability criteria: systolic blood pressure between 90-165 mmHg (mean arterial pressure 60–90 mmHg), heart rate between 58-110 beats per minute, respiratory rate less than 28 breaths per minute, fraction of inspired oxygen (FiO2) less than 60%, and positive end-expiratory pressure under 7.5 cm H2O. Furthermore, patients could not have experienced any new or increased doses of vasopressors or antidysrhythmics in the four hours leading up to the intervention. Those with a fever spike within the hour prior to the scheduled activity did not receive the intervention. Blood samples were collected daily for up to three contiguous days following a rest period and an ETM period, then weekly or on the day of ICU discharge (+24 hours). A lab technician, blinded to the intervention, analyzed the serum, while a trained RN, unaware of the ETM activity's frequency and in tensity, measured delirium and muscle strength during the ETM periods. |
